# Reevaluation of Performance of Electric Double-layer Capacitors from Constant-current Charge/Discharge and Cyclic Voltammetry

**DOI:** 10.1038/srep38568

**Published:** 2016-12-09

**Authors:** Anis Allagui, Todd J. Freeborn, Ahmed S. Elwakil, Brent J. Maundy

**Affiliations:** 1 Dept. of Sustainable and Renewable Energy Engineering, University of Sharjah, PO Box 27272, Sharjah, UAE; 2 Center for Advanced Materials Research, University of Sharjah, PO Box 27272, Sharjah, UAE; 3 Dept. of Electrical and Computer Engineering, University of Alabama, PO Box 870286, Tuscaloosa, USA; 4 Dept. of Electrical and Computer Engineering, University of Sharjah, PO Box 27272, Sharjah, UAE; 5 Nanoelectronics Integrated Systems Center (NISC), Nile University, Cairo, Egypt; 6 Dept. of Electrical and Computer Engineering, University of Calgary, Alberta, Canada

## Abstract

The electric characteristics of electric-double layer capacitors (EDLCs) are determined by their capacitance which is usually measured in the time domain from constant-current charging/discharging and cyclic voltammetry tests, and from the frequency domain using nonlinear least-squares fitting of spectral impedance. The time-voltage and current-voltage profiles from the first two techniques are commonly treated by assuming ideal *S*_*s*_*C* behavior in spite of the nonlinear response of the device, which in turn provides inaccurate values for its characteristic metrics. In this paper we revisit the calculation of capacitance, power and energy of EDLCs from the time domain constant-current step response and linear voltage waveform, under the assumption that the device behaves as an equivalent fractional-order circuit consisting of a resistance *R*_*s*_ in series with a constant phase element (CPE(*Q, α*), with *Q* being a pseudocapacitance and *α* a dispersion coefficient). In particular, we show with the derived (*R*_*s*_, *Q, α*)-based expressions, that the corresponding nonlinear effects in voltage-time and current-voltage can be encompassed through nonlinear terms function of the coefficient *α*, which is not possible with the classical *R*_*s*_*C* model. We validate our formulae with the experimental measurements of different EDLCs.

The soaring demand for portable consumer electronic products and alternative energy vehicles created a unique market place for electrochemical energy storage in double-layer capacitors (EDLC). EDLCs are known for their high power density and high degree of reversibility, an energy density that bridges the gap between conventional electrostatic/electrolytic capacitors and rechargeable batteries, and long-term self-discharge, while remaining low cost and environmentally compatible devices^1^. Unlike batteries and fuel cells that harvest the energy stored in chemical bonds through faradic reactions, the outstanding properties of EDLCs are principally the result of the nanometer-sized electrostatic charge separation at the interface between the large surface area porous electrode material and the electrolyte^2^. However, Chmiola *et al*. showed that sub-nanometric pore size smaller than the solvated ions (such as carbide-derived carbon) can drastically increase the energy being stored in the device^3^, which challenges the widely accepted traditional charge storage mechanism^4^,^5^. It has also been proven that the hybrid configuration, in which the electrodes are made out of porous carbon material for surface-based double-layer capacitance combined with battery material for volume-based pseudocapacitance, is an effective approach to enhance the energy density of the storage devices^6^,^7^,^8^,^9^,^10^,^11^. Thus, because the energy and power performance of EDLCs are determined by the capacitance of its electrodes, and the ionic and electronic charge transports in the cell^12^, most of the research studies are focused on the rational design and optimization of nanostructured materials, electrolytes, and auxiliary components^1^,^3^,^11^,^13^,^14^.

Notwithstanding, in contrast with conventional capacitors that have been available for over a century, the measurement methods for determining the main metrics of EDLCs, i.e. capacitance, internal resistance, stored energy and power, are still not properly standardized^15^,^16^. As a result, we see that the estimation of such parameters from the commonly employed steady-state and impulse electroanalytical techniques^15^,^16^,^17^,^18^ are most of the time adapted from the formulae used for ideal capacitors. For instance, with galvanostatic charge/discharge, which consists of studying the transient voltage response of the device when a stepping current I_cc_ is applied, the average capacitance is usually calculated from ref. 15:


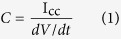


with *dV*/*dt* being the slope of the time-voltage curve. The capacitance is typically measured from the response of the device under different values of I_cc_. With cyclic voltammetry (CV) experiment, the current is recorded vs. a linearly changing cell voltage between the two terminals of the device, giving qualitative and quantitative information about the electrode processes. The integral capacitance *C* of the target electrode can be calculated from the CV curves as^18^:


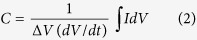


with the numerical integration of the current being over the half-cycle potential window (Δ*V*), and *dV*/*dt* is the voltage scan rate^15^. Since *C* depends on the sweep rates in the CV test, the device is usually charged and discharged at different rates and thus at different powers^19^. The stored energy is estimated from the dc capacitance and the voltage window as *E* = *C*Δ*V*^2^/2, and the power from the ohmic drop as *p* = *V*^2^/4*R*_*s*_, by assuming an ideal capacitive behavior^11^.

However, from the very specific name of EDLC devices (lossy capacitors, leaking capacitors, or pseudocapacitive devices), it is actually misleading to assume their ideality which is what is being done when using equations 1 and 2. In the frequency domain, EDLC devices exhibit constant phase or fractional power characteristics different from the response of ideal capacitors^20^. In Fig. 1(a) we show the Nyquist plot representation of impedance spectroscopy (EIS) for two EDLCs subject of this study, i.e. a Cooper Bussmann PowerStor supercapacitor (denoted PS, rated as 2.7 V with 3 F nominal capacitance and 0.060 Ω maximum equivalent series resistance (ESR) at 1 kHz) and a NEC/TOKIN supercapacitor (denoted NEC, rated as 5.5 V with 1 F nominal capacitance and 65 Ω maximum ESR at 1 kHz). The impedance response of the PS device is typical for an equivalent series resistance (*R*_*s*_) in series with a constant-phase element (CPE) behavior^21^,^22^,^23^,^24^,^25^,^26^, as it consists of a straight line of slope 13.17 Ω/Ω (i.e. 85.6°) with *R*_*s*_ = 40 mΩ (Im(*Z*) = 0) at 5.3 kHz. Complex nonlinear least-squares fitting of the impedance response using the model *R*_*s*_–CPE (*Z*_CPE_ = 1/*Qs*^*α*^ in which the pseudocapacitance *Q* is in units of F s^*α*−1^, *s* = *jω*, and the dispersion coefficient *α* can take on values between 1, for an element acting as an ideal capacitor, to 0, for a resistor^24^) resulted in (*R*_*s*_, *Q, α*) = (50 mΩ, 2.04 F s^*α*−1^, 0.95). Although *α* is very close to one, the device cannot be considered ideal and energy dissipation is expected. The impedance response of the NEC EDLC, on the other hand, shows a nonlinear behavior with more deviation from ideality, including a depressed semi-circle of 3.44 Ω diameter representing, a 43.0°-inclined pseudo-Warburg region (824 Hz to 28 mHz), and a quasi-vertical line of 85.4° inclination vs. the real axis in the low frequency region 28 mHz to 5 mHz. The *R*_*s*_-CPE fitting plot to the experimental data from 824 Hz to 5 mHz, and its parameters are shown in Fig. 1, although a double-dispersion model with two CPEs would be a better fit^11^. The dispersion coefficient *α* is found to be 0.70, further away from ideal capacitor. The Bode diagrams, i.e. phase angle shift of impedance vs. frequency, of both PS and NEC EDLCs are plotted in Fig. 1(b), and from which one can deduct more detail on the electric behaviors of the devices. The capacitive behavior manifests itself only close to the dc limit where the phase angle vs. log(|*f*|) tends to −90°. The PS EDLC shows a close-to-ideal capacitance over a wider frequency range. In an intermediary frequency region that spans a few decades, the devices show a tendency towards a resistive behavior with loss of capacitance, as the porous electrode material is not allowed to be fully charged.

Now giving that in the frequency domain EDLCs exhibit constant phase behavior, it is incorrect to revert to *R*_*s*_*C* modeling for the analysis of their behavior in the time domain^20^. Furthermore, although EIS is a powerful characterization tool to evaluate the equivalent electric circuits of dynamic processes, frequency response analysis remains quite expensive from hardware and software viewpoints. In this study, we show how to accurately characterize an EDLC device by extracting its equivalent circuit element parameters from the time domain constant-current charge/discharge responses and cyclic voltammetry using fractional-order calculus. We adopted the *R*_*s*_−CPE(*Q, α*) model which offers one extra degree of freedom when compared to the commonly used *R*_*s*_*C* model to better accommodate the response of the device. Our results on the time-domain responses of EDLCs have the merit to (i) provide additional and inexpensive alternatives to the standard EIS technique, while (ii) redefining the metrics of these devices (capacitance, power and energy) that are commonly and wrongly adopted from *R*_*s*_*C* models.

## Constant-Current Charging/Discharging

When the impedance *Z(s*) = *R*_*s*_ + 1/*Qs*^*α*^ of an equivalent electric model *R*_*s*_–CPE(*Q, α*) is excited by an input current step of amplitude I_cc_ > 0 (note that the same analysis below can be applied for a constant-current discharging step), the output voltage is given by:


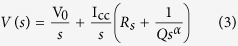


where V_0_ is the initial voltage. *V(s*) can be re-arranged to:


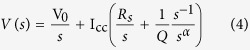


We now apply the inverse Laplace transform to equation 4 using the formula^27^:


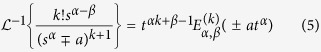


where *E* is the Mittag-Leffler function. Noting that *k* = 0, *a* = 0, and *β* = *α* + 1, this yields the time domain equation:


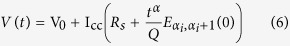


in which 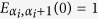, which reduces to the voltage-time characteristics of an *R*_*s*_–CPE equivalent model as:


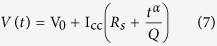


We verify that for an ideal capacitor (i.e. *α* = 1 and *Q* = *C* in Farads) we have the linear relationship:


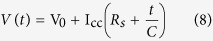


which leads to the capacitance expression in equation 1 by setting *R*_*s*_ = 0. Note that an effective capacitance^28^ in units of Farad can be defined for an EDLC by equating equations 7 and 8 giving:


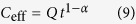


**Figure 1:**
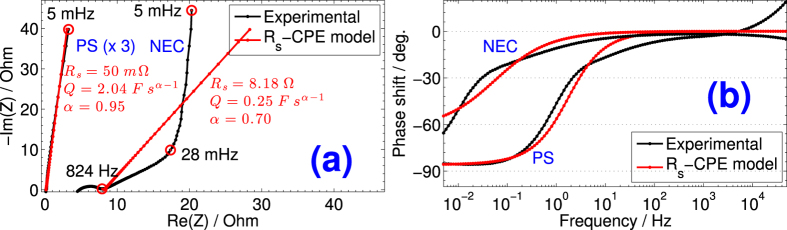
(**a**) Nyquist representation of impedance and (**b**) Bode diagrams for the NEC and PS EDLCs. Plots and parameters using complex nonlinear least-squares fitting to *R*_*s*_–CPE(*Q, α*) model are also shown.

**Figure 2:**
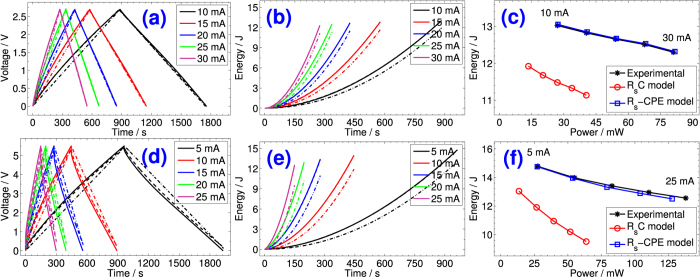
Constant-current time-voltage (**a**) and (**d**), time-energy (**b**) and (**e**), and power-energy (**c**) and (**f**) profiles of PS and NEC EDLCs respectively. The solid lines represent the experimental data, whereas the dash-dot lines and the dashed lines represent the fitted data using the *R*_*s*_*C* model and the *R*_*s*_–CPE(*Q, α*) model, respectively.

Figure 2(a) and (d) show in solid lines the fifth cycles of voltage-time response (the first few cycles are usually disregarded because of undefined initial conditions) collected at different dc charge/discharge currents for the PS and NEC EDLCs, respectively. All plots are practically symmetric showing first a steep increase of voltage due to dissipation in the equivalent series resistance *R*_*s*_, followed by a second quasi-linear stage corresponding to the charging of the pseudocapacitive material of the device. The discharge curves are also nonlinear by exhibiting first a quick voltage drop that increases with the increase of current rate, followed by a nonlinear capacitive region until zero voltage. These nonlinearities in voltage-time curves are characteristic features of the electric behavior of porous electrode capacitors^29^, that can not be properly captured by an *R*_*s*_*C* model. The deviation from ideality can also be demonstrated from the discrete Fourier transform analysis of harmonics obtained from the decomposition of current waveforms, as shown in Fig. S1. In particular, for NEC (see Fig. S1(c) and (d) showing the spectral amplitude of measured charging and discharging current signals respectively), the non-fundamental components extending to a few tens of mHz contribute with relatively important weights (vs. the magnitude of the fundamental harmonic) to the overall signals. This is most noticeable with the increase of current charge/discharge rates (see frequency response from ±25 mA waveforms). The effect of these frequency components of the power system harmonics can be demonstrated in connection with the Nyquist plot of impedance shown in Fig. 1(a). Around the knee frequency of *ca*. 28 mHz the impedance of NEC changes from the pseudo-Warburg impedance ((*R*_*s*_, *Q, α*) = (8.06 Ω, 0.15 F s^*α*−1^, 0.47) over the frequency range 28 mHz–824 Hz) to a close-to-ideal capacitive behavior ((*R*_*s*_, *Q, α*) = (16.6 Ω, 0.56 F s^*α*−1^, 0.93) over the frequency range 5 mHz–28 mHz). This knee frequency is one of the harmonics resulting from the Fourier transform analysis shown in Fig. S1(c) and (d), which means that the electric characteristics of the device extracted from galvanostatic charge/discharge measurements are averaged integral values over the frequency domain that take into account the contributions of both capacitive and pseudo-Warburg regions. When the knee frequency is within the interval set for fitting in the Nyquist plot, we clearly see that both *Q* and *α* decrease consequently, *e.g*. (*R*_*s*_, *Q, α*) is equal to (16.6 Ω, 0.56 F s^*α*−1^, 0.93) over the frequency range 5 mHz–28 mHz, (9.62 Ω, 0.29 F s^*α*−1^, 0.74) over the range 5 mHz–824 Hz, and (8.18 Ω, 0.25 F s^*α*−1^, 0.70) over the range 5 mHz–3 MHz (note that the device is not well fitted with the model of a series resistance associated with a CPE over extended ranges of frequency). This effect is not taken into account when the simple *R*_*s*_*C* model is used to analyze the voltage-time profiles of charging/discharging EDLCs.

We show in Fig. 2(a) and (d) using dash-dot lines the fitted data with equation 8 (the average capacitance values are shown in the second column of Tables S1 and S2 for PS and NEC EDLCs respectively, which were calculated from the slopes of the charge and discharge time-voltage curves using equation 1 and in which it is implicitly assumed that *R*_*s*_ = 0). The deviation from the experimental data is more pronounced for the NEC device characterized by a lower dispersion coefficient. In dashed lines we show the time-voltage plots of charging and discharging using equation 7, in which the parameters *R*_*s*_, *Q*, and *α* (summarized in Tables S1 and S2) were extracted from the experimental data using nonlinear least-squares fitting with optimization search for global minimum. It is clear that the *R*_*s*_–CPE (*Q, α*)-modeled data for both PS and NEC are in excellent agreement with the nonlinear behavior of the experimental charging/discharging of the devices. In contrast with the *R*_*s*_*C* model, the *R*_*s*_–CPE model takes into account the nonlinear behavior of an EDLC through the dispersion coefficient *α* which cannot be one.

From equation 7, we derive the following expressions for the power of an EDLC:


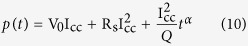



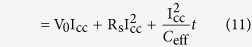


The energy is therefore given by:


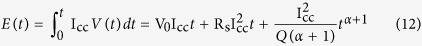



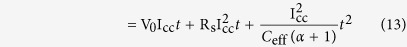


For *V*_0_ = 0, *R*_*s*_ = 0 and *α* = 1, we verify that *E(t*) = *q*^2^/2*C* = *CV*^2^/2 (*q* is the stored charge in Coulomb). The accumulated energy under different constant-current charge/discharge rates for both PS and NEC devices are plotted in Fig. 2(b) and (e), respectively. The experimental and modeled data using *R*_*s*_*C* and *R*_*s*_–CPE are shown in solid, dash-dot, and dashed lines, respectively. In contrast with the *R*_*s*_*C*-modeled data, the *R*_*s*_–CPE model shows excellent agreement with the experiment as a consequence of the proper calculation of the effective capacitance of the devices given by *C*_eff_ = *Qt*^1−*α*^, and by taking into account their dispersion coefficients (see computed values in Tables S1 and S2). In particular, the dependence of maximum power vs. maximum energy (*i.e*. Ragone plot, using the expression of *C*_eff_ with *t* = t_ss_ the time needed for full charge (or full discharge)) which is plotted in Fig. 2(c) and (d) for PS and NEC respectively, shows a much better agreement with the measurements for the *R*_*s*_–CPE-model when compared to the commonly used *R*_*s*_*C* model.

## Cyclic Voltammetry

In this section we revisit the calculation of the metrics of an EDLC from its charging with constant positive *dV*/*dt*. The same procedure can be followed for discharging to simulate the EDLC dynamics in CV experiment. Consider first the general case of an applied voltage *v(t*) across an *R*_*s*_–CPE(*Q, α*) model. The voltage *v(t*) can be written as:


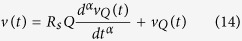


in which *v*_*Q*_(*t*) is the voltage across the pseudocapacitance *Q*. Applying the Laplace transform yields:


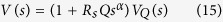


Thus, for *v(t*) = V_cc_*t*/t_ss_; 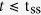 (V_cc_ is the steady-state upper limit of the linear voltage scan that will be reached at time *t* = t_ss_), we obtain:


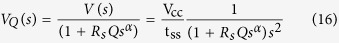


With *a* = 1/*R*_*s*_*Q*, we can write:


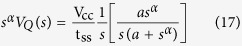


Using the inverse Laplace transform we get:


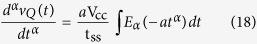


where *E* is again the Mittag-Leffler function. Now if we define *a* = *b*^*α*^ (i.e. *b* = (1/*R*_*s*_*Q*)^1/*α*^), then for significantly small *R*_*s*_ such that *b* → ∞, it is possible to write the following expansion^30^:


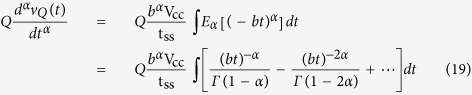


where *Γ*( · ) is Euler’s gamma function. The left-hand side of equation 19 is equal to the current *i(t*) = *dq(t*)/*dt*, which after integration with respect to time must be equal to the charge on a CPE alone if *R*_*s*_ → 0 (i.e. *b* → ∞):


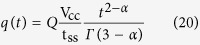


Note that at steady-state the charge 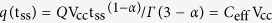, where *C*_eff_ is an effective capacitance in units of Farad defined as:


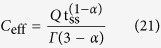


Then the current can be found to be:


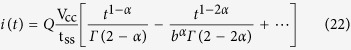



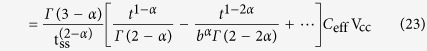


We verify that for an ideal capacitor, i.e. *R*_*s*_ = 0, *α* = 1 and *Q* = *C*, the current-time relationship given by equation 23 reduces to *i(t*) = *C*V_cc_/t_ss_. It is worth mentioning here that the effective capacitance is a quantity that depends on the way the EDLC device has been excited (compare equations 9 and 21) and eventually on the electric model being used.

**Figure 3:**
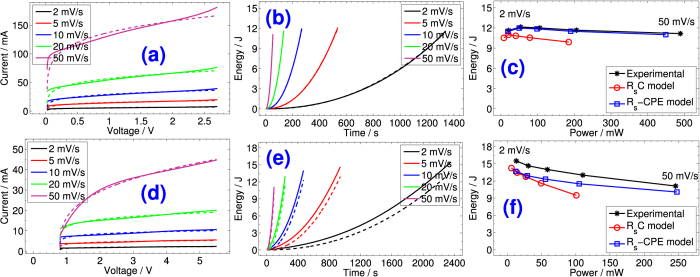
Current-voltage (**a**) and (**d**), time-energy (**b**) and (**e**), and power-energy (**c**) and (**f**) profiles at linear voltage scans for PS and NEC EDLCs respectively. The solid lines represent the experimental data, and the dashed lines represent the fitted data using *R*_*s*_–CPE(*Q, α*) model.

In Fig. 3(a) and (d), we show in solid lines the fifth half-cycles of current-voltage responses measured at different positive voltage scan rates (i.e. 2, 5, 10, 20, and 50 mV s^−1^) for PS and NEC EDLCs respectively. The voltammograms are close, but not ideally rectangular in shape, which is characteristic of non-ideal capacitors (for pure voltage-independent capacitive behavior, the current is linear with the voltage sweep rate and the voltammograms are ideal rectangles with mirror-image symmetry with respect to the zero current axis^31^). The increase of distortion, i.e. increase of positive slope of voltammetric current responses, is more noticeable for NEC which exhibits further deviation from ideality (Fig. 3(d)), and increases with the increase of voltage sweep rate for both devices due to the porous nature of the electrode material and the manifestation of more resistive behavior^31^. This is also due, as discussed in the previous section, to the composition of Fourier spectral response of the used voltage waveforms as shown in Fig. S2. Specifically for NEC (see Fig. S2(b)), it is clear that the 50 mV/s excitation has components below and above the knee frequency of 28 mHz, which means that the voltammetric response is a weighted average of low frequency capacitive behavior and medium to high frequency pseudo-Warburg behavior.

In the same figures (i.e. Fig. 3(a) and (d)), we show using dashed lines the current-voltage relationship expressed by equation 22 using four terms (with *v(t*) = V_cc_*t*/t_ss_; 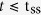). The (*R*_*s*_, *Q, α*) fitting parameters are summarized in Tables S3 and S4 for NEC and PS, respectively. It is clear that our model successfully accounts for the nonlinear behavior of the current which was not possible with the classical *R*_*s*_*C* circuit models based on the simple assumption that *i* = *CdV*/*dt*^31^,^32^,^33^. Other authors have simulated the CV measurements of EDLCs using the classical or modified Poisson–Nernst–Planck (PNP) models^12^,^34^,^35^, but in general these microscopic models still suffer from several limitations and assumptions for their validity, such as point charges instead of finite size of ions, or a maximum allowed ion concentration, or a maximum allowed potential^19^. Although in many recent papers attempts have been made to account for such limitations, the use of PNP-based models to characterize EDLCs is unlikely in practical situations. In contrast, our *R*_*s*_–CPE -based model (equation 21) represents a simple holistic description of the device that encompasses the nonlinear behavior of the current-voltage profiles obtained from CV tests. The average capacitances calculated from equations 2 and 21 under the different scan rates are shown Tables S3 and S4 for PS and NEC EDLCs respectively. The *R*_*s*_*C* capacitance is overestimated by *ca*. 2% versus the one estimated from the *R*_*s*_–CPE model for PS, which exhibits a close-to-ideal capacitive behavior, whereas it is as high as 10% in average for NEC EDLC. This discrepancy is not acceptable for practical design applications, which why our model is recommended instead.

From equation 22, we derive the power of an EDLC as:


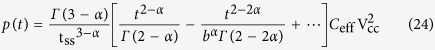


and the stored energy as:


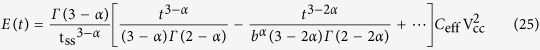


At steady-state, this energy is equal to:


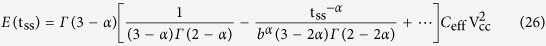


which, if *R*_*s*_ → 0, reduces to 
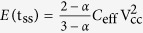
. The latter gives 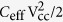 for *α* = 1 as expected. Assuming two terms only in equation 26 we obtain


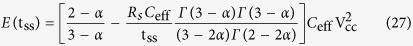


from which it is clear that the existence of *R*_*s*_ reduces the amount of energy stored in an EDLC. To minimize this effect, it is required to minimize the ratio *R*_*s*_*C*_eff_/t_ss_, which is equivalent to choosing a charging time t_ss_ such that:


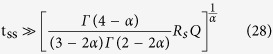


In Fig. 3(b) and (c) we show respectively the energy-time profiles at 2, 5, …, 50 mV/s scan rates, and the Ragone plot for the PS device. Our derived expressions are in excellent agreement with the experiment. The deviation from the measurements is more noticeable for the NEC device as shown in Fig. 3(e) and (f), but still the *R*_*s*_–CPE model is more reliable than the commonly used *R*_*s*_*C* model.

## Conclusions

In summary, we showed from the analysis of harmonics resulting from the fast Fourier transform of constant-current charge/discharge of an EDLC, and its frequency-dependent impedance that the time-voltage relationship can not be analyzed with the assumption of ideal *R*_*s*_*C* behavior as is commonly done. Instead, using the fractional-order *R*_*s*_–CPE (*Q, α*) model, we showed excellent agreement with the experimental time-voltage responses using the derived equation *V(t*) = I_cc_(*R*_*s*_ + *t*^*α*^/*Q*). The term *t*^*α*^ accounts for the nonlinear current evolution that the standard *R*_*s*_*C* model, giving by the expression *V(t*) = V_0_ + I_cc_(*R*_*s*_ + *t*/*C*), fails to simulate. An effective capacitance computed as *C*_eff_ = *Qt*^1−*α*^ in proper units of Farad is proposed, and from which the power (equation 11) and energy (equation 13) characteristics of the device can be calculated. In particular we showed that the commonly used formula for the stored energy, i.e. *q*^2^/2*C*, has to be replaced by *q*^2^/[*C*_eff_(*α* + 1)]. A recapitulation of the different expressions for calculating the metrics of an EDLC from galvanostatic charging using *R*_*s*_-CPE model are compared to those using *R*_*s*_*C* in Table 1.

In the same way, from the expression of current through a fractional-order capacitance given by *i(t*) = *Qd*^*α*^*v(t*)/*dt*^*α*^ instead of *i(t*) = *C* *dv(t*)/*dt* which is valid for ideal capacitance only, we derived the time-current relationship of an EDLC subjected to charging by linear voltage sweep (equation 23). Subsequently, we showed excellent agreement with the experiment of the voltage-current, time-energy, and power-energy profiles. The time domain expressions for CV profiles derived from the commonly used *R*_*s*_*C* model and the ones we propose here using the *R*_*s*_–CPE are also summarized in Table 1.

## Methods

Two commercial supercapacitors were selected for this study: a Cooper Bussmann PowerStor (denoted PS) carbon aerogel supercapacitor (part # HV0820-2R7305-R, rated as 2.7 V with 3 F nominal capacitance and 0.060 Ω maximum equivalent series resistance (ESR) at 1 kHz) and a NEC/TOKIN (denoted NEC) supercapacitor (part #FGR0H105ZF, rated as 5.5 V with 1 F nominal capacitance and 65 Ω maximum ESR at 1 kHz). All measurements were carried out by using a BioLogic VSP-300 electrochemical workstation at room temperature.

## Additional Information

**How to cite this article**: Allagui, A. *et al*. Reevaluation of Performance of Electric Double-layer Capacitors from Constant-current Charge/Discharge and Cyclic Voltammetry. *Sci. Rep.* **6**, 38568; doi: 10.1038/srep38568 (2016).

**Publisher's note:** Springer Nature remains neutral with regard to jurisdictional claims in published maps and institutional affiliations.

## Supplementary Material

Supporting Information

## Figures and Tables

**Table 1:** Recapitulative table of the different metrics of EDLC computed from galvanostatic charge and linear voltage sweep using the standard *R*_*s*_ *C* model and the *R*_*s*_–CPE model.

		*R*_*c*_*C* model (with *R*_*s*_ = 0 and V_0_ = 0)	*R*_*s*_–CPE model
Galvanostatic charge (I_cc_ > 0)	Voltage/V	*V(t*) = V_0_ + I_cc_(*R*_*s*_ + *t*/*C*) = I_cc_*t*/*C*	*V(t*) = V_0_ + I_cc_(*R*_*s*_ + *t*^*α*^/*Q*)
	Current/A	*i* = I_cc_	*i* = I_cc_
	Capacitance/F	*C* = I_cc_t_ss_/V_cc_	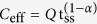 *
	Power/W	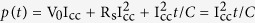	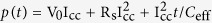
	Energy/J	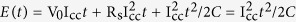	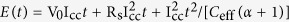
Cyclic voltammetry (forward voltage sweep)	Voltage/V	V_cc_*t*/t_ss_	V_cc_*t*/t_ss_
	Current/A	*i* = *C*V_cc_/t_ss_	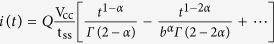 **
	Capacitance/F	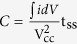	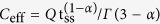
	Power/W	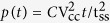	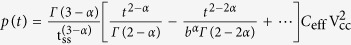 **
	Energy/J	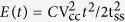	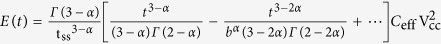 **

^*^Q = I_cc_*t*^*α*^/V_cc_ in F s^*α*−1^.

^**^b = (1/*R*_*s*_*Q*)^1/*α*^.
