# Corrigendum: Reevaluation of Performance of Electric Double-layer Capacitors from Constant-current Charge/Discharge and Cyclic Voltammetry

**DOI:** 10.1038/srep46828

**Published:** 2017-06-19

**Authors:** Anis Allagui, Todd J. Freeborn, Ahmed S. Elwakil, Brent J. Maundy

Scientific Reports 6: Article number: 3856810.1038/srep38568; published online: 12 09 2016; updated: 06 19 2017

The original version of this Article contained errors. In the Abstract,

“The time-voltage and current-voltage profiles from the first two techniques are commonly treated by assuming ideal *S*_*s*_*C* behavior in spite of the nonlinear response of the device, which in turn provides inaccurate values for its characteristic metrics”.

now reads:

“The time-voltage and current-voltage profiles from the first two techniques are commonly treated by assuming ideal *R*_*s*_*C* behavior in spite of the nonlinear response of the device, which in turn provides inaccurate values for its characteristic metrics”.

In addition, a factor was omitted for 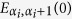 in the ‘Constant-Current Charging/Discharging’ section where:


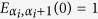


now reads:


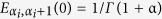


As a result, the values of Q in Tables S1 and S2 of the Supplementary Information were incorrect and have now been corrected.

The factor Q in Equations 7, 9, 10 and 12, and in the Voltage/V and Capacitance/F expressions for Galvanostatic charge (I_cc_ > 0) in the R_s_-CPE model in Table 1 has been replaced with Q*Γ*(1 + α).

In the ‘Constant-Current Charging/Discharging’ section,

“In contrast with the *R*_*s*_*C*-modeled data, the *R*_*s*_–CPE model shows excellent agreement with the experiment as a consequence of the proper calculation of the effective capacitance of the devices given by *C*_eff_ = *Qt*^1−*α*^, and by taking into account their dispersion coefficients (see computed values in Tables S1 and S2)”.

now reads:

“In contrast with the *R*_*s*_*C*-modeled data, the *R*_*s*_–CPE model shows excellent agreement with the experiment as a consequence of the proper calculation of the effective capacitance of the devices given by *C*_eff_ = *QΓ(1* + *α)t*^1−*α*^, and by taking into account their dispersion coefficients (see computed values in Tables S1 and S2)”.

In the ‘Conclusion’ section,

“Instead, using the fractional-order *R*_*s*_–CPE (*Q, α*) model, we showed excellent agreement with the experimental time-voltage responses using the derived equation *V(t*) = I_cc_(*R*_*s*_ + *t*^*α*^/*Q*). The term *t*^*α*^ accounts for the nonlinear current evolution that the standard *R*_*s*_*C* model, giving by the expression *V(t*) = V_0_ + I_cc_(*R*_*s*_ + *t/C*), fails to simulate. An effective capacitance computed as *C*_eff_ = *Qt*^1−*α*^ in proper units of Farad is proposed, and from which the power (equation 11) and energy (equation 13) characteristics of the device can be calculated”.

now reads:

“Instead, using the fractional-order *R*_*s*_–CPE (*Q, α*) model, we showed excellent agreement with the experimental time-voltage responses using the derived equation *V(t*) = I_cc_(*R*_*s*_ + *t*^*α*^/*QΓ*(1 + α)). The term *t*^*α*^ accounts for the nonlinear current evolution that the standard *R*_*s*_*C* model, giving by the expression *V(t*) = V_0_ + I_cc_(*R*_*s*_ + *t/C*), fails to simulate. An effective capacitance computed as *C*_eff_ = *QΓ*(1 + α)*t*^1−*α*^ in proper units of Farad is proposed, and from which the power (equation 11) and energy (equation 13) characteristics of the device can be calculated”.

These errors do not affect the findings of the paper and have now been corrected in the PDF and HTML versions of this Article, and in the accompanying Supplementary Information.

