# Supplementary material for: Reevaluation of Performance of Electric Double-layer Capacitors from Constant-current Charge/Discharge and Cyclic Voltammetry
Source: Sci Rep. 2016 Dec 9;6:38568. doi: 10.1038/srep38568 (PMC5146949; doi:10.1038/srep38568)
Supplement: Supporting Information [file srep38568-s1.pdf]

Reevaluation of Performance of Electric Double-layer Capacitors from Constant Current Charge/Discharge and Cyclic Voltammetry

Anis Allagui[1,2,*], Todd J. Freeborn[3], Ahmed S. Elwakil[4,5], and Brent J. Maundy[6]

[1]Dept. of Sustainable and Renewable Energy Engineering, University of Sharjah, PO Box 27272, Sharjah, UAE
[2]Center for Advanced Materials Research, University of Sharjah, PO Box 27272, Sharjah, UAE
[3]Dept. of Electrical and Computer Engineering, University of Alabama, PO Box 870286, Tuscaloosa, USA
[4]Dept. of Electrical and Computer Engineering, University of Sharjah, PO Box 27272, Sharjah, UAE
[5]Nanoelectronics Integrated Systems Center (NISC), Nile University, Cairo, Egypt
[6]Dept. of Electrical and Computer Engineering, University of Calgary, Alberta, Canada
[*]aallagui@sharjah.ac.ae

SUPPORTING INFORMATION

FFT of Constant-current Charge/Discharge and Linear Voltage Waveforms

We used the fast Fourier transform (FFT) algorithm of Matlab for the computation of estimate component frequencies in the discrete data of:

1. current charge and discharge in the galvanostatic test (see figure S1), and
2. voltage scan waveform in the voltammetric test (see figure S2).

The window length was set to 2048 data points.

Calculation of EDLCs Metrics from R_s-CPE Model

The nonlinear least-squares minimization for data fitting was carried out using Matlab's lsqcurvefit function with Trust-Region-Reflective (TRR) algorithm. The fitting routine attempts to numerically solve the problem:

$$\min_x \ f_0(x) = \sum_{i=1}^{n} \left[f(x_i; R_s, Q, \alpha) - y_i \right]^2 \ ; \quad R_s, Q, \alpha > 0 \tag{S1}$$

where (R_s, Q, α) constitutes the set of characteristic parameters to minimize the function $f_0(x)$, $f(x_i; R_s, Q, \alpha)$ is the fitting function (equation 7 for constant-current charge/discharge and equation 22 for linear voltage sweep) evaluated at x_i, y_i is the measured response at x_i, and n is the total number of collected data points. A constraint is added to the problem to limit the possible solutions for the resistance and pseudocapacitance to real positive values. Also, a negative value of the dispersion coefficient α indicates inductive characteristics which is not considered here. The code was configured to use Matlab's MultiStart with 50 iterations to find the global solution of (R_s, Q, α).

The extracted values of (R_s, Q, α) satisfying equation 7 for galvanostatic charge/discharge are shown summarized in tables S1 and S2 for PS and NEC EDLCs respectively. Similarly, the extracted values of (R_s, Q, α) satisfying equation 22 for linear voltage scans are shown summarized in tables S3 and S4 for PS and NEC EDLCs respectively. The average capacitance from the R_s–CPE (denoted C_{eff}) is compared with the average capacitance calculated from the standard R_sC model for both techniques.

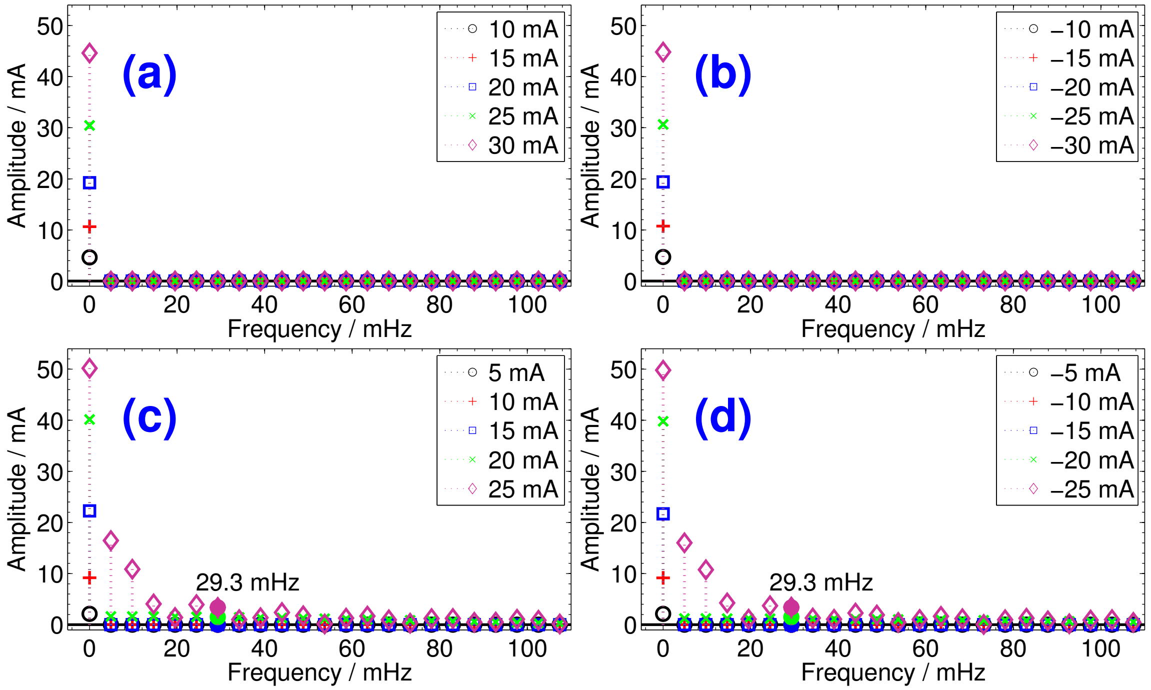

Figure S1. Amplitude spectrum of current waveforms calculated using discrete Fourier transform over a window of 2048 points for PS ((a) & (b) for charge/discharge) and NEC ((c) & (d) for charge/discharge) ECDLs

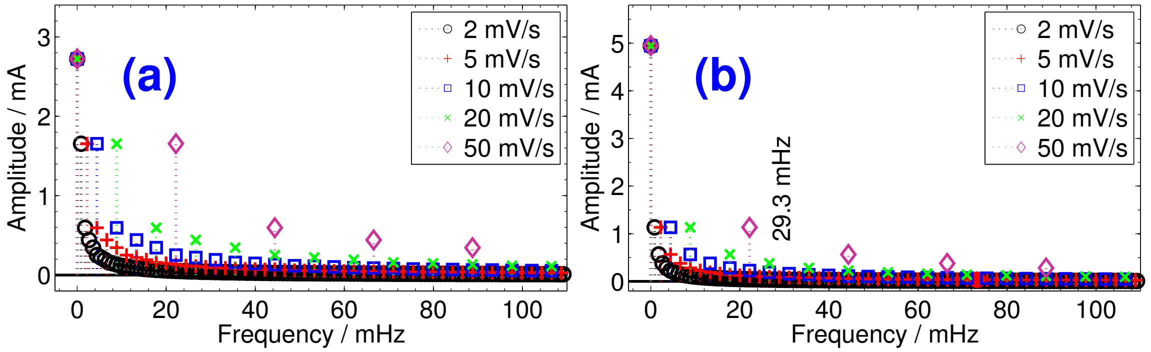

Figure S2. Amplitude spectrum of voltage waveforms calculated using discrete Fourier transform over a window of 2048 points for PS (a) and NEC (b) ECDLs during linear scan voltammetry

Table S1. Extracted set of parameters (R_s, Q, α) from ±10, 15, … 30 mA charge/discharge waveforms of the PS EDLC using nonlinear least-squares optimization with equation 7. The average capacitance from the R_s–CPE (C_{eff}, calculated using eq. 9) is compared with the average capacitance calculated from the standard R_sC model (eq. 1) over (i) the full voltage window and (ii) 20 to 80% the nominal voltage

	R_sC model (eq. 1)		R_s-CPE model (eq. 7)				
I_{cc} / mA	C / F	C (20-80%) / F	R_s / Ω	Q / F s$^{\alpha-1}$	α	Norm of the Residual	C_{eff} (eq. 9) / F
10.00	3.27	3.31	0.00	1.11	0.84	0.87	3.25
15.00	3.21	3.25	0.00	1.10	0.83	0.65	3.18
20.00	3.15	3.19	0.00	1.11	0.83	0.57	3.13
25.00	3.11	3.15	0.00	1.12	0.83	0.53	3.08
30.00	3.06	3.09	0.00	1.15	0.83	0.45	3.03
-10.00	3.23	3.33	0.82	3.30	1.00	2.73	3.30
-15.00	3.18	3.28	1.08	3.24	1.00	1.21	3.24
-20.00	3.13	3.22	0.97	3.19	1.00	0.67	3.19
-25.00	3.09	3.18	0.82	3.15	1.00	0.43	3.15
-30.00	3.05	3.13	0.68	3.10	1.00	0.30	3.10

Table S2. Extracted set of parameters (R_s, Q, α) from ±5, 10, … 25 mA charge/discharge waveforms of the NEC EDLC using nonlinear least-squares optimization with equation 7. The average capacitance from the R_s–CPE (C_{eff}, calculated using eq. 9) is compared with the average capacitance calculated from the standard R_sC model (eq. 1) over (i) the full voltage window and (ii) 20 to 80% the nominal voltage

	R_s-C model (eq. 1)		R_s-CPE model (eq. 7)				
I_{cc} / mA	C / F	C (20-80%) / F	R_s / Ω	Q / F s$^{\alpha-1}$	α	Norm of the Residual	C_{eff} (eq. 9) / F
5.00	0.89	0.90	0.00	0.26	0.82	2.91	0.88
10.00	0.83	0.86	10.10	0.31	0.83	1.00	0.85
15.00	0.79	0.82	12.79	0.33	0.84	0.53	0.82
20.00	0.76	0.80	13.53	0.33	0.84	0.45	0.80
25.00	0.73	0.77	13.46	0.33	0.83	0.51	0.78
-5.00	0.90	0.96	20.64	0.28	0.83	4.60	0.93
-10.00	0.84	0.90	19.42	0.28	0.81	1.43	0.88
-15.00	0.81	0.87	17.33	0.28	0.80	0.85	0.86
-20.00	0.78	0.83	15.97	0.27	0.79	0.73	0.83
-25.00	0.75	0.79	14.89	0.27	0.78	0.76	0.81

Table S3. Extracted set of parameters (R_s, Q, α) using nonlinear least-squares optimization with equation 22 from linear scan voltammetry test of the PS EDLC. The average capacitance from the R_s–CPE (C_{eff}, calculated using eq. 21) is compared with the average capacitance calculated from the standard R_sC model (eq. 2)

	R_s-C model (eq. 2)	R_s-CPE model (eq. 22)				
Scan rate / mV s^{-1}	C / F	R_s / Ω	Q / F s$^{\alpha-1}$	α	Norm of the Residual	C_{eff} (eq. 21) / F
2	2.89	0.00	0.70	0.79	0.00	2.87
5	3.01	0.00	0.86	0.78	0.00	2.99
10	2.97	0.00	0.95	0.78	0.00	2.95
20	2.89	0.00	1.12	0.79	0.01	2.88
50	2.71	0.00	1.42	0.81	0.06	2.78

Table S4. Extracted set of parameters (R_s, Q, α) using nonlinear least-squares optimization with equation 22 from linear scan voltammetry test of the NEC EDLC. The average capacitance from the R_s–CPE (C_{eff}, calculated using eq. 21) is compared with the average capacitance calculated from the standard R_sC model (eq. 2)

	R_s-C model (eq. 2)	R_s-CPE model (eq. 22)				
Scan rate / mV s^{-1}	C / F	R_s / Ω	Q / F s$^{\alpha-1}$	α	Norm of the Residual	C_{eff} (eq. 21) / F
2	0.94	0.00	0.29	0.85	0.00	0.85
5	0.89	0.00	0.34	0.86	0.00	0.80
10	0.84	0.00	0.36	0.87	0.00	0.77
20	0.77	0.00	0.34	0.85	0.00	0.71
50	0.63	0.62	0.17	0.69	0.00	0.59
